# Cavity‐dwelling “acorn ants” nest in aborted red pine cones

**DOI:** 10.1002/ecy.70583

**Published:** 2026-11-05

**Authors:** Nathan L. Haan, Elizabeth C. Tolrud, Julian R. Dupuis, Brianna Gidley, Anna Grotelueschen, Cameron Hay, Simon Janevic, Adyaan Menon, Skyler Umney

**Affiliations:** ^1^ Department of Entomology University of Kentucky Lexington Kentucky USA; ^2^ University of Michigan Biological Station, University of Michigan Pellston Michigan USA

**Keywords:** ant, cavity nesting, facilitation, habitat selection, seed predation

Cavity‐dwelling ants inhabit the litter layer of deciduous forest floors across Eastern North America and Europe. Often called “acorn ants,” species belonging to this consortium form small colonies in acorns, hickory nuts, hollow twigs, and similar confined spaces. Although there are multiple genera of cavity‐nesting ants, the best studied belongs to *Temnothorax* (Figure 1a). This is a diverse and globally distributed genus with a variety of nesting habits (Prebus, 2017), but in Eastern North America, they are best known for their habit of using hollow aboveground cavities. Acorn ants' housing arrangements often involve facilitation, with ants moving into abandoned cavities that other insects create. This nesting strategy can involve nut weevils (*Curculio* spp.) or other seed predators that oviposit in developing acorns; larvae consume the seed and bore an escape hole that will later serve as an ant colony entrance to the hollow nut (Creighton, 1950). Acorn ants can nest in a variety of hollow objects, but much of our knowledge of nesting strategies centers on how they interact with their eponymous housing structure. Therefore, we were surprised to find large numbers of acorn ants nesting in aborted cones belonging to red pine (*Pinus resinosa*), using galleries likely formed by the red pine cone beetle (*Conophthorus resinosae*; Figure 1b–e; Video S1). We first observed this at the University of Michigan Biological Station (UMBS), located on the northern tip of Michigan's lower peninsula, in July 2024 (N Haan, personal observation), and subsequently confirmed that use of pine cones is commonplace in several forest stands across northern Michigan in 2025–2026 (Appendix S1: Section S1). We noticed they used both cones and acorns (*Quercus rubra*) in locations where both cavities were available, but they were also common in forest stands containing red pines without oaks or other obvious cavity sources.

**FIGURE 1:**
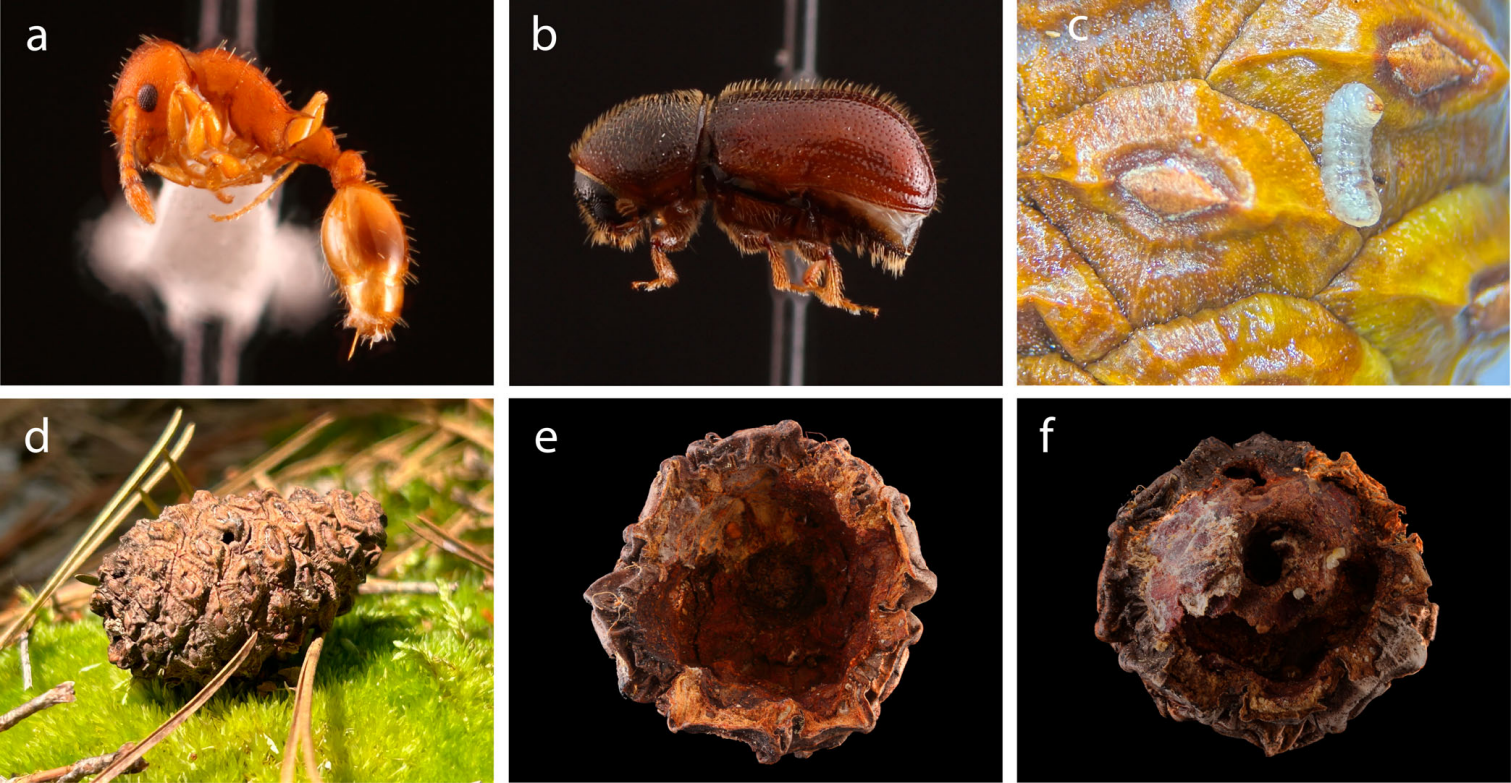
Overview of organisms involved in this study. (a) *Temnothorax ambiguus* worker; (b) adult *Conophthorus resinosae* collected from inside a red pine cone; (c) larval *C. resinosae* on a newly fallen cone (we removed it from the feeding gallery and placed on cone exterior); (d) aborted red pine cone on the forest floor with *C. resinosae* exit hole visible; (e) basal and (f) apical sections of an aborted cone that was excavated by *C. resinosae* and later occupied by *T. ambiguus*. Panels a, b, e, and f are z‐stacked images compiled in ZereneStacker 1.04; panels e and f were edited to remove halos and background objects. For a video of *T. ambiguus* in an opened cone, see Video S1. Photos by Nathan L. Haan.

The most common species we found nesting in cones was *Temnothorax ambiguus* (95% of occupied cones during systematic sampling, see Appendix S1: Section S1 for field survey methods and Appendix S1: Section S2 for DNA barcoding methods). *Temnothorax* in North America is small‐bodied ants that make colonies of just a few dozen workers. The whole superorganism can fit in a single cavity or may use a network of cavities on the forest floor (Herbers, 1986; Herbers & Stuart, 1996). Acorn ants are frequent house‐hunters, navigating a shifting landscape of ephemeral resources. New potential nests drop from the canopy each year, but as they decompose, there is pressure to vacate them and find suitable replacements. The ants can be limited by social parasites, nest site availability, and space on the forest floor, and often compete within and between species for suitable cavities and/or territory (Herbers, 1986; Prather et al., 2018). The habits of acorn ants have long intrigued naturalists, and since their nests are small and easy to access, they are a popular study system that has helped us learn about diverse ecological topics such as competition, social parasitism, thermal adaptation and overwintering biology, collective decision‐making, and colony structure of social insects (Foitzik et al., 2004; Herbers, 1986, 1989; Herbers & Foitzik, 2002; Herbers & Stuart, 1996; Karlik et al., 2016; M. Herbers & A. Johnson, 2007; Prather et al., 2018; Pratt et al., 2002; Pratt & Pierce, 2001; Prileson et al., 2023; Varoudis et al., 2018; Yan & Prebus, 2024).

All the ant‐inhabited cones we found appeared to have been excavated by the red pine cone beetle, *C. resinosae* (Curculionidae, subfamily Scolytinae; Figure 1b,c). Red pine cones take two summers to mature; *C. resinosae* attack them in large numbers in late spring and early summer during the cone's second year of development (Lyons, 1956; Mattson, 1978, 1980). This seed predator specializes in red pine and its congener jack pine, *Pinus banksiana* (de Groot & Borden, 1991). It can cause devastating seed crop losses; Mattson (1978) collected data from eight red pine stands in Michigan, Wisconsin, and Minnesota and found >80% of seeds were destroyed by insects, with *C. resinosae* responsible for large proportions of the loss. In line with this, we often found densities of >10 aborted cones per m^2^ under red pines in our study area (Figure 2; methods in Appendix S1: Section S1).

**FIGURE 2:**
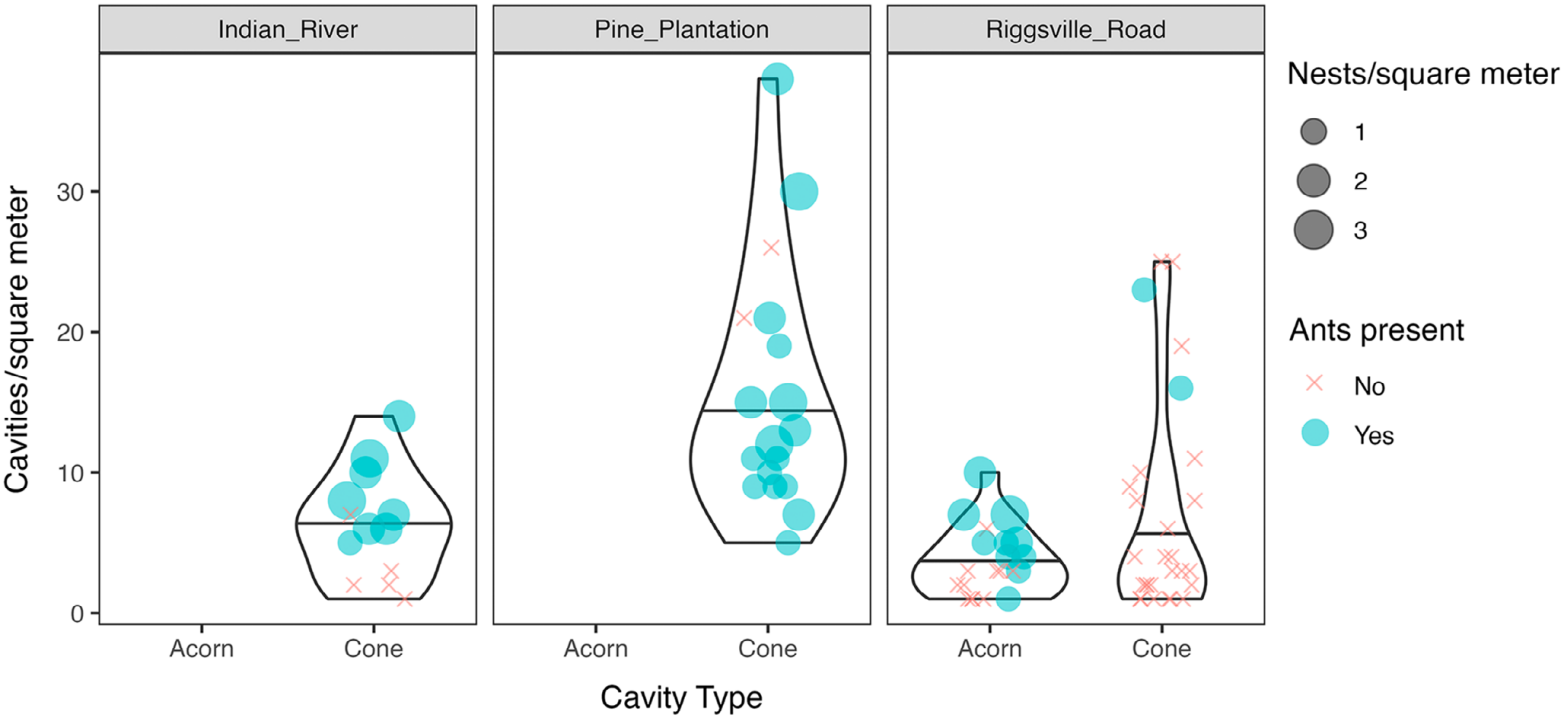
Density of cavities (acorn or cone) and cavity‐nesting ants on the forest floor at three study sites (See Appendix S1: Table S1 for site details). Violin plots depict cavity densities within 80 1‐m^2^ quadrats distributed across the three sites; horizontal lines correspond to medians. The points comprising each distribution are jittered and sized in proportion to the number of ant nests found within the quadrat (range: 0–3); quadrats with no nests present for that cavity type are depicted with an × rather than a point.

Female *Conophthorus* adults burrow into the cone base, working toward the apex within or alongside the central axis. After ovipositing, they exit and plug the basal hole with debris and resin, sealing the chamber (Lyons, 1956). Damage to the cone axis usually severs its vascular connection to the tree, causing it to abort and fall to the forest floor during summer without opening. Larvae expand the oviposition gallery, eating the developing seeds and some of the surrounding tissues, and eventually bore exit holes on the side of the cone (Figure 1d). The other primary seed predator of *P. resinosa* is the Pyralid moth *Dioryctria disclusa*; however, this species does not cause the cone to fully abort without opening and therefore seems unlikely to produce ant‐ready cavities (Mattson, 1986). A sealed cone can contain up to 10 *Conophthorus* larvae (Lyons, 1956); we often observed 1–5 larvae per cone at our study sites, where aborted cones were actively falling in June and July (N Haan personal observation). Newly fallen cones usually contained immature and/or adult stages of *C. resinosae*. In a few instances, we found *Temnothorax* cohabitating with *Conophthorus* larvae in newly fallen cones, although the nature of this relationship is unknown. More often we found ants in cones that appeared to have fallen at least one year previous (i.e., they were brown and desiccated rather than partially green and resinous).

We found high densities of cone‐nesting ants in several forest stands in northern Michigan (Figure 2; Methods in Appendix S1: Section S1). We surveyed ant colonies systematically in 2025 at three forest stands, where we found there was often one or more nests per m^2^ beneath red pine trees (range 0–3, Figure 2). We sampled 637 cavities, including 560 cones and 77 red oak acorns, inventoried in 80 1‐m^2^ quadrats distributed across the three sites. Figure 2 shows the density of cones, acorns, and ant nests within each of them. Cone lengths ranged from 10 to 60 mm (mean 26 mm), and larger cones were more likely to contain ant nests (binomial generalized linear models [GLMs], likelihood‐ratio test *p* = 0.004; Appendix S1: Figure S2).

Colony composition for *T. ambiguus* in cones was similar to that reported in other studies. The number of queens per nest ranged from 0 to 3 (mean = 0.83); previous work on *Temnothorax* has shown they often have more than one queen per colony (polygyny) and that a single colony may utilize a network of nest sites simultaneously (polydomy), leading to cavities that contain workers and brood but no queen (Alloway et al., 1982; Herbers, 1986, 1989). Therefore, some of the nests we sampled within a given quadrat belonged to the same colony. The mean numbers of workers and brood (larvae + pupae) per occupied cone were 12.60 and 27.00, respectively.

One of the forest stands we sampled in 2025 contained a mix of red pines and red oaks; here we found very few cone‐nesting ants (mean 0.07 nests/m^2^, Figure 2). Instead, acorn‐based nests were >10× more common (0.71 nests/m^2^, Figure 2), even though there were usually twice as many cones available as acorns in a sampling quadrat (7.04 cones/m^2^ compared to 3.68 acorns on average). We also observed that acorns were more variable in terms of which species they contained; out of the 11 ant‐inhabited acorns we found, only 5 contained *T. ambiguus* while the others contained *Brachymyrmex depilis* (2) or *Tapinoma sessile* (4). This led us to ask if, given the choice, *T. ambiguus* prefers to use acorns.

We conducted behavioral assays in which *T. ambiguus* colonies chose between acorns and cones (see Appendix S1: Section S3). In contrast to our field observations, these trials suggested they do not strongly prefer one type of cavity over the other. In 2025, we ran choice trials using 15 colonies that had been nesting in pine cones. We evicted each group from their cone and placed the full colony in an arena surrounded by 9 aborted cones and 9 hollow acorns, giving them 48 h to relocate (Appendix S1: Figure S3). The nest types they chose were precisely an even split: six groups moved into an acorn (or acorns), six groups used only cones, and three used a combination of both. Then, in 2026, we repeated this protocol with 20 additional colonies, half of which had been nesting in acorns and the other half in pine cones, in case their natal cavity type might influence preference. Again, there was limited evidence for innate preference. Among colonies that had been nesting in acorns, four selected an acorn, five selected a cone, and one scattered, failing to relocate. For colonies that had been nesting in cones, three moved into an acorn, five chose a cone, one selected both, and one failed to relocate. Overall, the mismatch between our field observations and behavioral assays suggests preference does not center on cavity type per se, but on other factors. These could include cavities' internal architecture, moisture, microbial communities, size and quantity of openings, competition, or food availability on the surrounding forest floor (e.g., Karlik et al., 2016; Pratt & Pierce, 2001). It is also possible that foundresses choose based on different criteria than workers.

Cavity‐nesting ants provide a useful window into ant ecology and evolution because they are easy to access, observe, and manipulate. Landscapes where they use cones, acorns, or a combination of nest types could provide leverage to ask new questions or revisit old ones. For example, the demographic and population dynamics of masting oaks, red pines, and their respective seed predators control the availability of each cavity type. How might these factors conspire to shape ant communities on forest floors, and the ecological functions they carry out? Several more specific questions involve the interplay between nesting preference, fitness outcomes, and competition. Does preference vary between populations, and how might this map out across *Temnothorax* species ranges and those of the cavities they use (see range map, Appendix S1: Figure S1)? If ants are restricted to one cavity type or the other, do fitness outcomes or overwintering success differ? Given that competition for space and nest sites can be intense, could nest types mediate niche partitioning or character displacement?

It is well known that acorn ants nest in a variety of substrates other than acorns. For example, in *The Ants of North America*, Creighton (1950) reports a personal observation that *T. ambiguus* nests mostly in open soil rather than cavities. The newly described *T. caryaluteus*, which was previously classified as *T. ambiguus*, is arboreal and nests in dead branches (Prebus et al., 2024), and in general, *Temnothorax* in the Northeast United States can build nests in soil, in hollow stems, under bark, and under rocks (Ellison et al., 2012). There may be a wealth of unexplored phenomena related to cavity‐nesting ants' diverse housing arrangements, especially as they play out across landscapes and species' ranges. Future studies involving acorn ants could embrace this diversity and leverage it to learn new things about insect ecology.

## CONFLICT OF INTEREST STATEMENT

The authors declare no conflicts of interest.

## Supporting information


Appendix S1.

Video S1.

## Data Availability

Data and code used to generate figures (Haan et al., 2026) are available in Zenodo at https://doi.org/10.5281/zenodo.22881364.

## References

[ecy70583-bib-0001] Alloway, T. , R. Stuart , C. Thomas , A. Buschinger , and M. Talbot . 1982. “Polygyny and Polydomy in Three North American Species of the Ant Genus Leptothorax Mayr (Hymenoptera: Formicidae).” Psyche: A Journal of Entomology 89: 249–274. 10.1155/1982/64124.

[ecy70583-bib-0002] Creighton, W. S. 1950. The Ants of North America, Vol. 104. Cambridge, MA: Bulletin of the Museum of Comparative Zoology.

[ecy70583-bib-0003] de Groot, P. , and J. H. Borden . 1991. “Life History of the Jack Pine Tip Beetle, *Conophthorus banksianae* Mcpherson (Coleoptera: Scolytidae): Implications for Species Status.” The Canadian Entomologist 123(1): 211–217. 10.4039/Ent123211-1.

[ecy70583-bib-0024] Ellison, A. M. , N. J. Gotelli , E. J. Farnsworth , and G. D. Alpert . 2012. A Field Guide to the Ants of New England, 398 pp. New Haven, CT: Yale University Press.

[ecy70583-bib-0004] Foitzik, S. , V. L. Backus , A. Trindl , and J. M. Herbers . 2004. “Ecology of Leptothorax Ants: Impact of Food, Nest Sites, and Social Parasites.” Behavioral Ecology and Sociobiology 55(5): 484–493. 10.1007/s00265-003-0718-9.

[ecy70583-bib-0005] Haan, N. , E. Tolrud , J. Dupuis , B. Gidley , A. Grotelueschen , C. Hay , S. Janevic , A. Menon , and S. Umney . 2026. “Cavity‐Dwelling “Acorn Ants” Nest in Aborted Red Pine Cones [Dataset].” Zenodo. 10.5281/zenodo.22881364. PMC13740656 42989900

[ecy70583-bib-0006] Herbers, J. M. 1986. “Nest Site Limitation and Facultative Polygyny in the Ant Leptothorax Longispinosus.” Behavioral Ecology and Sociobiology 19(2): 115–122. 10.1007/BF00299946.

[ecy70583-bib-0007] Herbers, J. M. 1989. “Community Structure in North Temperate Ants: Temporal and Spatial Variation.” Oecologia 81(2): 201–211. 10.1007/BF00379807. 28312539

[ecy70583-bib-0008] Herbers, J. M. , and S. Foitzik . 2002. “The Ecology of Slavemaking Ants and Their Hosts in North Temperate Forests.” Ecology 83(1): 148–163. 10.1890/0012-9658(2002)083[0148:TEOSAA]2.0.CO;2.

[ecy70583-bib-0009] Herbers, J. M. , and R. J. Stuart . 1996. “Multiple Queens in Ant Nests: Impact on Genetic Structure and Inclusive Fitness.” The American Naturalist 147(2): 161–187. 10.1086/285845.

[ecy70583-bib-0010] Karlik, J. , M. J. Epps , R. R. Dunn , and C. A. Penick . 2016. “Life Inside an Acorn: How Microclimate and Microbes Influence Nest Organization in Temnothorax Ants.” Ethology 122(10): 790–797. 10.1111/eth.12525.

[ecy70583-bib-0011] Lyons, L. A. 1956. “Insects Affecting Seed Production in Red Pine: Part I.” The Canadian Entomologist 88(10): 599–608. 10.4039/Ent88599-10.

[ecy70583-bib-0012] M. Herbers, J. , and C. A. Johnson . 2007. “Social Structure and Winter Survival in Acorn Ants.” Oikos 116(5): 829–835. 10.1111/j.0030-1299.2007.15679.x.

[ecy70583-bib-0013] Mattson, W. J. 1978. “The Role of Insects in the Dynamics of Cone Production of Red Pine.” Oecologia 33(3): 327–349. 10.1007/BF00348117. 28309596

[ecy70583-bib-0014] Mattson, W. J., Jr. 1980. “Cone Resources and the Ecology of the Red Pine Cone Beetle, Conophthorus Resinosae (Coleoptera: Scolytidae).” Annals of the Entomological Society of America 73(4): 390–396. 10.1093/aesa/73.4.390.

[ecy70583-bib-0015] Mattson, W. J., Jr. 1986. “Competition for Food Between Two Principal Cone Insects of Red Pine, Pinus Resinosa.” Environmental Entomology 15(1): 88–92. 10.1093/ee/15.1.88.

[ecy70583-bib-0016] Prather, R. M. , K. Kozmary , and S. Powell . 2018. “Combat, Co‐Existence, and Resource Partitioning in Acorn‐Dwelling Temnothorax Ants.” Insectes Sociaux 65(3): 455–463. 10.1007/s00040-018-0634-7.

[ecy70583-bib-0017] Pratt, S. C. , E. B. Mallon , D. J. Sumpter , and N. R. Franks . 2002. “Quorum Sensing, Recruitment, and Collective Decision‐Making During Colony Emigration by the Ant Leptothorax Albipennis.” Behavioral Ecology and Sociobiology 52(2): 117–127. 10.1007/s00265-002-0487-x.

[ecy70583-bib-0018] Pratt, S. C. , and N. E. Pierce . 2001. “The Cavity‐Dwelling Ant Leptothorax Curvispinosus Uses Nest Geometry to Discriminate Between Potential Homes.” Animal Behavior 62: 281–287. 10.1006/anbe.2001.1777.

[ecy70583-bib-0019] Prebus, M. 2017. “Insights Into the Evolution, Biogeography, and Natural History of the Acorn Ants, Genus *Temnothorax* Mayr (Hymenoptera: Formicidae).” BMC Evolutionary Ecology 17: 250. 10.1186/s12862-017-1095-8. PMC5729518 29237395

[ecy70583-bib-0020] Prebus, M. , N. Nguyen , G. Doering , and D. Booher . 2024. “ *Temnothorax caryaluteus* sp. Nov. (Hymeoptera: Formicidae): A New Ant Species from the Eastern United States.” European Journal of Taxonomy 970: 175–202. 10.5852/ejt.2024.970.2757.

[ecy70583-bib-0021] Prileson, E. G. , J. Clark , S. E. Diamond , A. Lenard , O. A. Medina‐Báez , A. R. Yilmaz , and R. A. Martin . 2023. “Keep Your Cool: Overwintering Physiology in Response to Urbanization in the Acorn Ant, *Temnothorax curvispinosus* .” Journal of Thermal Biology 114: 103591. 10.1016/j.jtherbio.2023.103591. 37276746

[ecy70583-bib-0022] Varoudis, T. , A. G. Swenson , S. D. Kirkton , and J. S. Waters . 2018. “Exploring Nest Structures of Acorn Dwelling Ants with X‐Ray Microtomography and Surface‐Based Three‐Dimensional Visibility Graph Analysis.” Philosophical Transactions of the Royal Society, B: Biological Sciences 373(1753): 20170237. 10.1098/rstb.2017.0237. PMC6030587 29967301

[ecy70583-bib-0023] Yan, J. L. , and M. Prebus . 2024. “Variability of Nest Relocation Behavior Among Acorn Ant Species.” Insectes Sociaux 71(1): 49–60. 10.1007/s00040-024-00950-x.

