# Supplementary material for: Cavity‐dwelling “acorn ants” nest in aborted red pine cones
Source: Ecology. 2026 Nov 5;107(11):e70583. doi: 10.1002/ecy.70583 (PMC13740656; doi:10.1002/ecy.70583)
Supplement: Supplementary file 1 [file ECY-107-e70583-s002.pdf]

Appendix S1

Cavity-dwelling "acorn ants" nest in aborted red pine cones

Nathan L. Haan, Elizabeth C. Tolrud, Julian R. Dupuis, Brianna Gidley, Anna Grotelueschen, Cameron Hay, Simon Janevic, Adyaan Menon, Skyler Umney

Ecology

Section S1. Methods for field exploration of cavity use and colony characteristics

We used field surveys to explore the spatial distribution, density, and colony structure of cone- and acorn-inhabiting ants. We asked, (1) is the cone-dwelling habit more widespread than the immediate grounds at the University of Michigan Biological Station (UMBS) where we first observed it? (2) What is the density of ant nests in pine cones and acorns? (3) How are colonies distributed across cavities – are they polydomous and/or polygynous? (4) Where both pine cones and acorns are present, is there any evidence that they use one cavity type preferentially over the other?

To learn whether the cone-dwelling habit is commonplace (1), we explored several forest stands in Northern Michigan in 2025 and 2026 (Fig S1, Table S1). At each of these we haphazardly opened red pine cones to see if they housed *T. ambiguus*. If ants were not found on short order, we continued to open as many as 100 cones from across the forest floor until we found them. We found *T. ambiguus* in every forest stand we searched except one (Table S1).

To address questions 2-4 we sampled forest floors systematically at three locations. One was a mixed oak-pine forest stand on the west side of UMBS (45°33'5"N, 84°42'6"W). Another was an over-mature red pine plantation (45°36'44"N, 84°46'14"W; 9 km NW of the first site); this forest stand was dominated by red pine but contained a diverse understory with beech

(*Fagus grandifolia*), serviceberry (*Amelanchier* sp.), and an herbaceous layer dominated by bracken fern *(Pteridium aquilinum*). The third site was a natural forest stand containing red pine, white pine (*Pinus strobus*)*,* maple (*Acer* spp.) and an understory dominated by bracken fern (location: 45°21'43"N, 84°39'51"W; 22 km south of UMBS). At each site we haphazardly selected red pine trees. Below each tree, in places where at least one aborted cone could be observed from a standing position without manipulating the litter layer, we dropped 1 m^2 quadrats and searched the litter layer exhaustively. We counted the number of cones and acorns, recorded their length and width, and opened all of them to look for ants. Any cavities containing ants were sealed in falcon tubes and returned to the lab. For each of these we identified them to species, counted the number of mature queens, workers, alates, and brood. Summaries of colony composition are in main text and Figs 2, S2. Finally, we used binomial generalized linear models to ask if larger cones were more likely to be occupied. We compared model with a fixed term for cone length to an intercept-only model, using a likelihood ratio test. Adding the term for length significantly improved model fit over the baseline ($\Delta D = 8.39$, $p = 0.004$). Estimated log-odds of occupancy increased by 0.051 per mm of cone length.

Section S2. Molecular barcoding to confirm identity of *Temnothorax ambiguus*

We used DNA barcoding of *cytochrome oxidase subunit I* (COI) to confirm identities of *Temnothorax* ants. We followed manufacturer's recommendations unless otherwise noted. QIAGEN DNeasy extraction kits were used on single, whole ants and we eluted in 100 µL of buffer AE. Primers lco22me (5' GGTCAACAAATCATAAAGATATTGG 3') and hco700me (5' TCAGGGTGACCAAAAAATCA 3') were used in PCRs with New England Biolabs OneTaq 2x master mix and gel electrophoresis on a 1.5% gel was used to check PCR products before Sanger

Sequencing with Eurofins Genomics (Louisville, Kentucky). Chromaseq v1.71 implemented in Mesquite v4.01 (Maddison and Maddison 2025a,b) was used to assemble sequences with manual inspection of chromatograms. BLAST and BOLD searches were used to determine a molecular identity of these sequences, and a representative sequence was uploaded to GenBank (accession PX491252).

Section S3. Methods for assessing cavity type preference

We conducted behavioral assays by evicting cone-dwelling *T. ambiguus* colonies and allowing them to choose between relocating to cones or acorns. In 2025, we collected 15 colonies from cones we sourced from 5 locations on the grounds surrounding research buildings and classrooms at UMBS. The minimum criterion for including a colony in the assay was for it to contain at least one queen, 10 workers, and some amount of brood. The number of workers in colonies we used ranged from ca. 10-40, although we did not count them at the outset because they were active and our priority was to transfer them to the choice arenas. Since *T. ambiguus* is both polygynous and polydomous, it is possible that some of the 'colonies' we tested were part of the same supercolony, and we did not control for this except by searching for cones within 5 locations with red pine stands that were >100 m apart. While searching for cones, we broke them open and if they appeared to contain an ant nest we moved them to a falcon tube, dissected under a microscope, and transferred them to choice arenas. We started the assays on 29 July 2025 and ran them for 48 h.

Choice arenas were glass culture dishes (d = 20 cm; h = 8 cm) with exposed masking tape lining the upper lip to prevent escape (Fig S3). To moderate moisture levels and create an environment that mimics the forest floor, we placed a layer of moss in each arena with a small

amount of soil and water at the bottom. We collected the moss from near the shore of Douglas Lake, a few meters from the location where we had first observed cone-nesting ants. The moss also contained decomposing leaf fragments and pine needles. We collected the cones and acorns for use in the trial concurrently with the ant colonies, from the same general locations. For a cone to be included in the trial, it needed to have the characteristic appearance of being aborted, i.e., it was in arrested development and most or all the scales had failed to dehisce. These cones always have an entrance hole at their base (although it may be plugged) and usually 1 or more exit holes toward the middle, plus occasional openings where scales have begun to dehisce or sutures released in early stages of decomposition. For red oak acorns, the acorn had to be at least partially hollow and have either a very narrow crack and/or an exit hole from a weevil. We discarded acorns with holes larger than ~2.5 mm and any that were not intact or had wide cracks. Before trials, we placed all cavities in a -80 C freezer for 18 h to kill any ants that might already be inhabiting them. We included 9 acorns and 9 pine cones in each arena (Fig S3). We included this large number because ants presumably choose cavities based on several criteria that were not feasible to measure without dissecting the cavities – for instance, moisture levels, aperture sizes, the state of galleries inside the cavity, microbial communities, etc. Rather than quantifying all of these variables, we furnished the arena with 9 of each cavity type so the ants could explore and choose, with the assumption that at least some of the cavities would meet their criteria.

After dissecting colonies under a microscope and ensuring they met criteria for the trials, we placed the entire colony and remnants of the nest cone we had destroyed in the center of an arena on a ~2x2 cm piece of office paper. We used the paper to keep eggs and brood from falling through the moss. Within a few hours, the ants had vacated the nest fragments. Workers pulled brood down into the moss, and we could observe scouts moving from cavity to cavity throughout

the arena. After 48 h we dissected all cavities and noted whether they were occupied. All 15 groups of ants we deployed in arenas successfully translocated to a new cavity (or cavities). Upon concluding the trials there were ≤2 previously-inhabited cavities per replicate with freeze-killed ants that had been incidentally collected; the ants in our trials avoided these cavities in all cases. Since the cavity types they selected were exactly an even split, we did not perform statistical analyses.

In 2026 we repeated this assay with 20 additional colonies. This time, we collected half of them from acorns and the other half from pine cones, in case their natal cavity type might influence their subsequent choices. To control all factors except natal cavity type, we collected these ants from the same mixed oak-pine forest stand as described in S1, where we found them in both cavity types but disproportionately in acorns. Choice trials took place from June 17-19. Methods were the same as the previous assay except we used 6 cavities of each type instead of 9. Out of the 20 groups of ants, 18 successfully relocated into new cavities. The other two failed to organize emigration and scattered without relocating brood or queens into either cavity type. Again, since there was again a near-even split in which cavity type they selected, we did not perform a statistical analysis. Results are described in the main text.

References

Maddison, D.R., & W.P. Maddison. 2025a. Chromaseq: a Mesquite package for analyzing sequence chromatograms. Version 1.71 https://chromaseq.mesquiteproject.org

Maddison, W. P. and D.R. Maddison. 2025b. Mesquite: a modular system for evolutionary analysis. Version 4.02. https://www.mesquiteproject.org

Petry, W., & S. Taylor. 2025. Clone of “Digital representations of tree species range maps from “Atlas of United States Trees” by Elbert L. Little, Jr. Retrieved from github.com/wpetry/USTreeAtlas. doi: 10.5281/zenodo/15468153

Prebus, M., Nguyen, N., Doering, G., Booher, D. (2024). *Temnothorax caryaluteus* sp. nov. (Hymeoptera: Formicidae): a new ant species from the Eastern United States. *European Journal of Taxonomy, 970*. https://doi.org/10.5852/ejt.2024.970.2757

Tables

Table S1. Locations for exploratory and systematic sampling in this study (see also Fig S1).

Coordinates	Site Name	Site characteristic	*T. ambiguus* nesting in cones?	Year(s) observed	Systematic sampling?
46°37'52"N 85° 3'7"W	Paradise	Red pine/jack pine barrens	Yes	2026	
46°15'35"N 85° 0'30"W	Trout Lake	Mixed pine/maple	Yes	2026	
45°36'44"N 84°46'14"W	Pellston Plantation	Overmature/abandoned red pine plantation	Yes	2025	X
45°33'5"N 84°42'6"W	UMBS Riggsville	Mixed pine/red oak	Yes	2025 2026	X
45°21'43"N 84°39'51"W	Indian River	Mixed pine/ hardwood	Yes	2025	X
45°28'57"N 84°13'28"W	Black Lake	Mixed pine/ hardwood	Yes	2025	
44°15'50"N 85°20'15"W	Cadillac	Mixed pine/aspen/hardwood	Yes	2026	
44°21'32"N 84°51'27"W	Reedsburg Dam	Mixed pine/hardwood	Yes	2026	
43°31'46"N 86° 7'22"W	Pine Point	Mixed pine/white oak	No	2025	

Figures

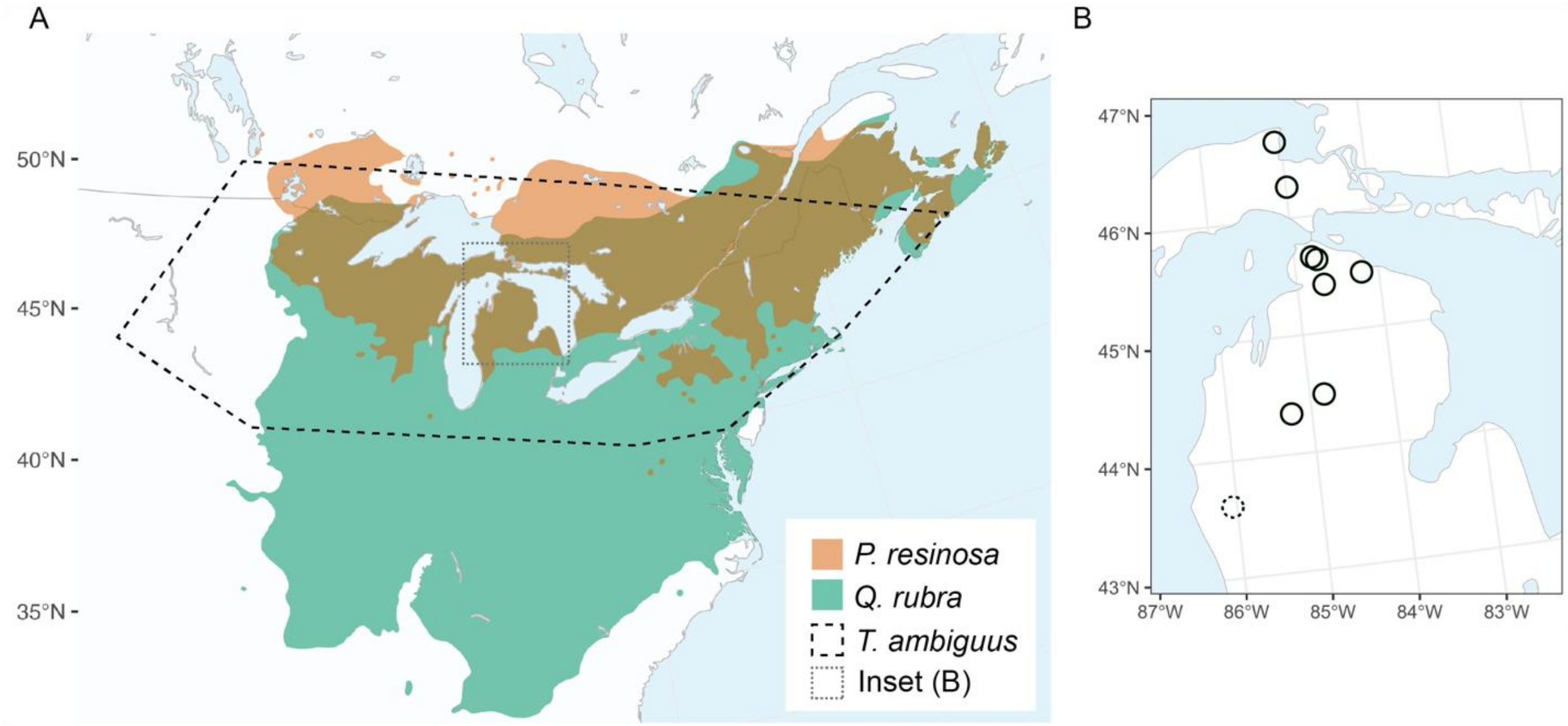

Figure S1. Map of relevant species ranges and study locations. A: Ranges of red pine (*Pinus resinosa*), red oak (*Quercus rubra*), and their overlap are shown by solid colors (data from Little's Atlas of US Trees, retrieved from Petry & Taylor 2025). The estimated range of *T. ambiguus* is shown with a dashed line. This is a hull approximated from Prebus (2024), who differentiated *T. ambiguus* from the newly-described *T. caryaluteus.* B: Locations in northern Michigan where we searched for *T. ambiguus* nests in red pine cones (see also Table S1). Solid circles indicate locations where *T. ambiguus* was present in cones; the dashed circle represents a location where aborted red pine cones were present but we did not detect *T. ambiguus* nesting in them.

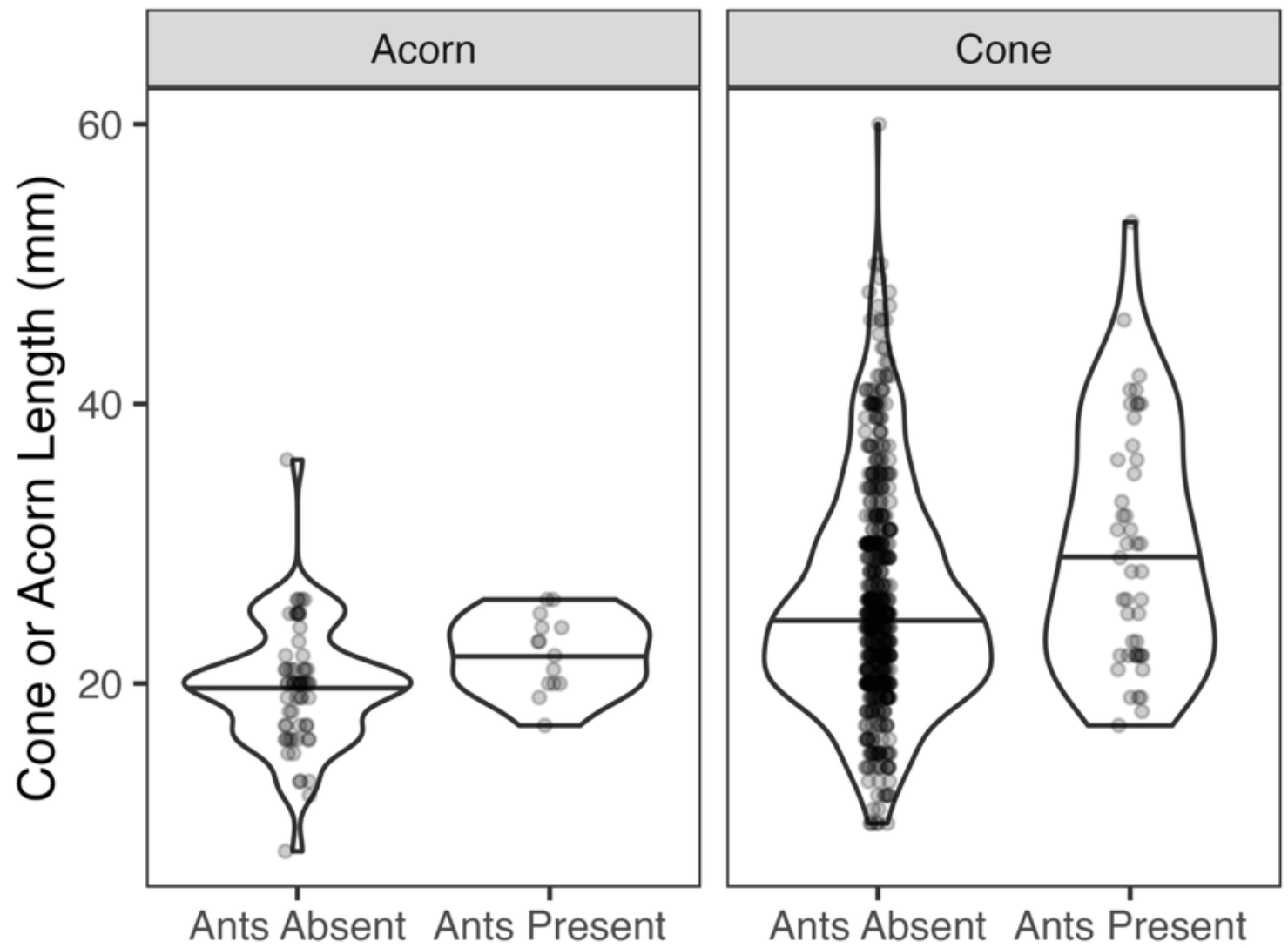

Figure S2. Larger cavities were more likely to contain ant colonies. Jittered points correspond to cone or acorn length (mm) in acorns or cones with or without ant colonies in them. Violin plots summarize data distributions.

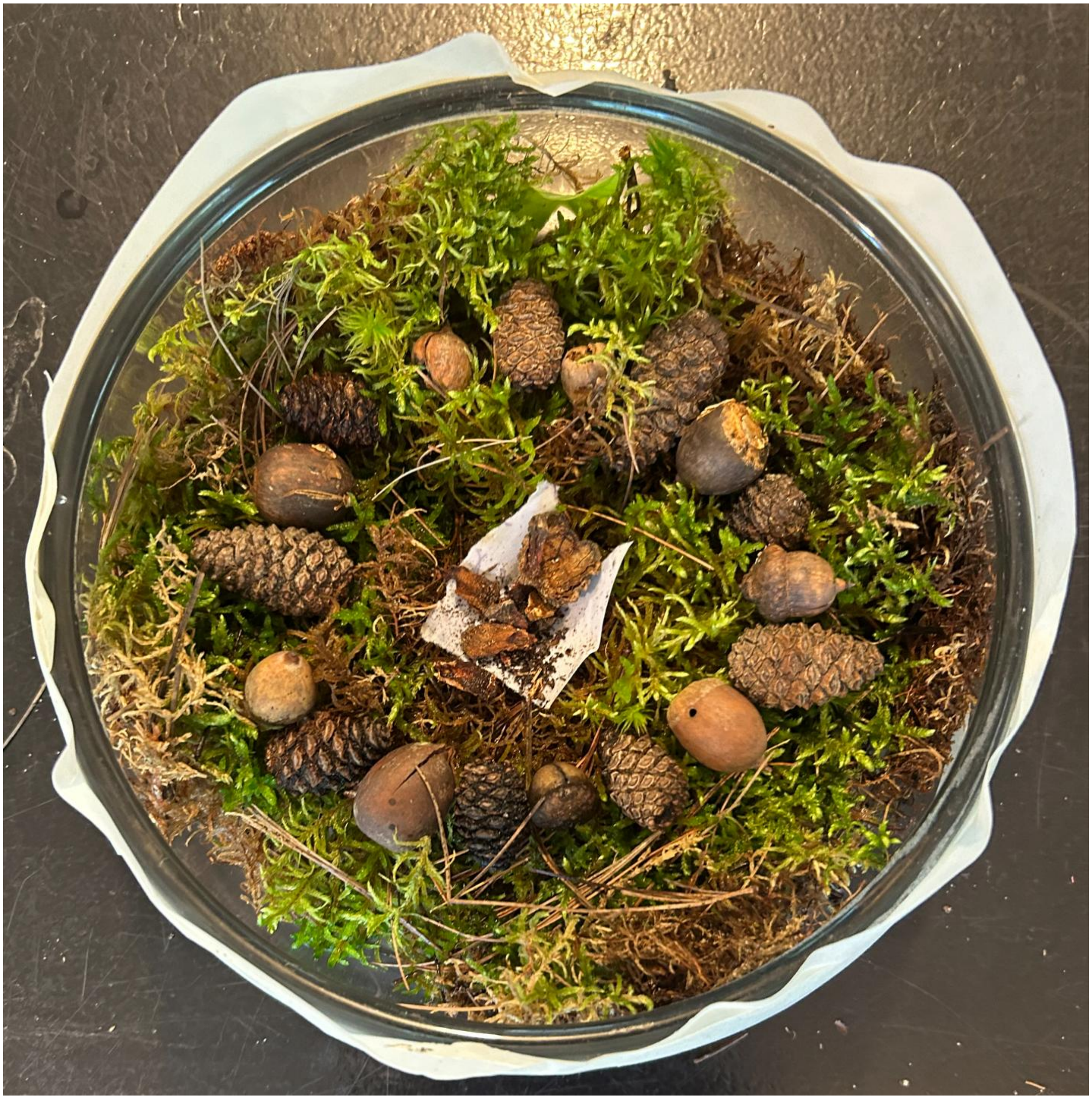

Figure S3. Example arena from choice trails in which *T. ambiguus* selected between cone or acorn cavity types. Photo by Nathan L. Haan.
